# Hospitalization for acute cerebellitis in children affected by varicella: how much does it cost?

**DOI:** 10.1186/s13052-020-00875-8

**Published:** 2020-08-06

**Authors:** Elena Bozzola, Stefano Guolo, Giulia Macchiarulo, Lidia Festa, Giulia Spina, Andrzej Krzysztofiak, Annalisa Grandin, Mauro Bozzola, Massimiliano Raponi, Alberto Villani

**Affiliations:** 1 Pediatric and Infectious Disease Unit, Bambino Gesù Children Hospital, Rome, Italy; 2 Medical Direction, Bambino Gesù Children Hospital, Rome, Italy; 3 University of Pavia, Pavia, Italy

**Keywords:** Chickenpox, Cost, Hospitalization, Children

## Abstract

**Background:**

Chickenpox is a highly contagious airborne disease caused by the varicella zoster virus. It is generally benign and self-limiting, but it may be responsible of life-threatening complications. Acute cerebellitis (AC) is the most common neurological complication and is associated with prolonged hospitalization in the acute phase (HAP).

**Aim of the study:**

To estimate the costs of AC HAP in children affected by varicella.

**Materials and methods:**

We retrospectively reviewed the medical records of a pediatric cohort hospitalized for chickenpox AC over a period of 15 years (from October 2003 to October 2018) and we analyzed acute care costs. For any patient the HAP has been calculated. The final value includes cost of hospital accommodation and management at the Pediatric and Infectious Diseases Unit. To this cost, the price of procedures (imaging, laboratory exams, medical and paramedical evaluations) and medical treatments was added.

**Results:**

In the study period, 856 children had been hospitalized for varicella. Out of them, 65 met a diagnosis of AC and were included in the study. The hospitalization length was of 10 days (range 3–20 days). The median cost of HAP for each patient was of 5366 euro, with an average annual cost of 23,252 €. The most significant part of HAP is due to the cost of hospital accommodation and management at the Pediatric Infectious Diseases Unit, which was about € 537.78 for a single day.

**Discussion:**

Although AC post-varicella is rare, its HAP cost is not negligible resulting in substantial economic burden. Vaccination would have probably prevented varicella and AC complication, avoiding hospitalization.

**Conclusions:**

Financial studies are important for evaluate the cost saving in order to influence public funding decisions. Further studies are necessary to investigate the economic burden of the disease.

## Background

Chickenpox is a highly contagious airborne disease caused by the varicella zoster virus (VZV). Even if it is considered benign and self-limiting, it may be responsible of complications and may require hospitalization [1]. In details, 0.1 to 1.5% of children affected by varicella are admitted to hospital due to a severe course of varicella [1–4]. Among neurological complications, varicella had been related to post infectious encephalitis, cerebellitis, acute myelitis and stroke or stroke like episodes, meningitis, encephalitis, myelitis and vasculopathy [5]. Acute cerebellitis (AC) is the most common neurological complication of chickenpox as it may represent the 44.7% of all neurological complications [5, 6]. Furthermore, it affects 0.05% of children with chickenpox infection [7]. In childhood, AC is associated with prolonged hospitalization in the acute phase (HAP). To date, vaccination constitutes the best preventive strategies against VZV infections [8].

### Aim of the study

Aim of the study is to estimate the costs of AC-HAP in children affected by varicella.

## Materials and methods

For the purpose of our study, we retrospectively reviewed the medical cards of patients hospitalized at Bambino Gesù Children Hospital, Rome, Italy, for chickenpox from October 2003 to October 2018. Patients over 18 years of age as well as children with immunodeficiency disorders were excluded. According to the literature, the diagnosis of chickenpox was clinical, based on the evidence of typical skin lesions in varying stages of development and resolution [9]. A neurological complication was defined as an unfavorable neurological evolution occurring within 3 weeks of varicella onset [10]. The diagnosis of AC was clinical as well, mainly based on the following findings: ataxia, unsteady gait or fine motor movement, trembling of the head and trunk in an upright position and the extremities when attempting to move against gravity [11]. The medical records of patients with the clinical-confirmed diagnosis. Patients who did not fulfill the inclusion criteria were excluded from the study. As for the others, direct medical costs were extracted from the Lazio Regional Health Service Tariffs. The appropriate procedure codes were applied, in order to evaluate the single cost of any exam and therapy. For any patient the HAP has been calculated. The final value includes cost of hospital accommodation and management at the Pediatric and Infectious Diseases Unit. To this cost, the price of procedures (imaging, laboratory exams, medical and paramedical evaluations) and medical treatments was added. HAP cost of patients with cerebellitis in varicella was compared to HAP cost of patients affected by varicella not complicated by AC. The t-test was used to compare the costs between the two groups.

## Results

We identified 856 children hospitalized for chickenpox. Among them, 181 (21%) were affected by neurological complications, mainly by AC (65 children, 7.59%). The mean age of patients affected by AC was of 5.49 years (range from 1.5 to 15.58 years, median 4.75 years). The proportion of male and female was similar (53.8% males and 46.2% female). Any patient had no underlying medical condition. None of the included patients had been previously vaccinated for varicella. The median cost of HAP for each patient was of 5366 euro (range from 1763 to 11,872 euro; mean cost of 6068 euro), higher than the one extracted by ICD-9-CM code from 052.0 to 052.9 (range from 1660 to 1800 euro). Medical condition both at AC onset and during hospitalization may have complicated the disease, requiring a prolonged length stay, further exams and therapies. We observed a similar trend of the mean HAP cost between patients aged from 1 to 5 years (30 patients), 5 to 10 years (28 patients) and older than 10 years (3 patients), respectively 6138, 6324 and 6430 euro.

Varicella therapy involves symptomatic care, pain management and medical treatment. For this reason, we evaluated for each patient the cost of laboratory exams (median cost of € 180,42; mean cost of € 154,21€; range from € 0 to € 498,64), medical and paramedical examinations (median cost of € 45,32; mean cost of € 45,45; range from € 20,66 to € 103,3) and therapeutic treatment (median cost of € 38,4; mean cost of € 42,42; range from € 15,36 to € 284,81). Drugs, such as antiviral or steroid treatment, contribute to higher HAP. In fact, thirty children (46%) who required a medical therapy had an HAP cost of 2757 euro higher than those without therapy.

Nevertheless, the most significant part of HAP is represented by the cost of hospital accommodation and management at the Pediatric Infectious Diseases Unit, which is about € 537.78 for a single day. Patients had been hospitalized for a mean time of 10.5 days (range from 3 to 20 days).

We compared the hospitalization cost between the 65 patients affected by varicella AC (Group A) and the other 791 patients admitted to the hospital for varicella but without AC (Group B) and hospitalized for a mean time of 7 days (range from 2 to 19 days).

HAP cost in Group A was higher compared Group B (€ 5366 and € 3806 respectively). In details, we found out a significant higher instrumental cost in Group A than in Group B (€ 143,76 and € 65,67 respectively), correlated to imaging exams.

Table 1 summarizes the HAP cost, the price of procedures (imaging, laboratory exams, medical and paramedical evaluations) and medical treatments in Groups A and B (Table 1).

**Table 1:** Comparison of HAP costs between patients affected by AC and those without AC

Parameters (median value)	Patients with AC	Patients without AC	p
Total cost (€)	5366	3806	0.59
Laboratory cost (€)	180,42	178,22	0.06
Instrumental cost (€)	143,76	65,67	0.02
Special visits (€)	45,32	42,61	0.09
Therapy costs (€)	38,4	28,53	0.06

## Discussion

Chickenpox is a childhood vaccine-preventable disease which may require hospitalization and relevant medical care services, representing a financial cost [12].

There is evidence that varicella has a significant economic impact.

We performed a MEDLINE search to estimate the cost of AC HAP, using the keywords ((“Chickenpox”[Mesh]) AND “Chickenpox/complications”[Mesh]) AND (“Chickenpox/economics”[Mesh]). Our review concerned scientific publications in the time period between September 2009–September 2018, referring to patients younger than 18 years of age, without chickenpox but not cerebellar complications 2) if they concerned cerebellitis but not cost analysis 3) if they concerned patients older than 18 years. Our MEDLINE analysis identified 6 reports. Nevertheless, after application of exclusion criteria, none of them remained for review. They were excluded because: concerned costs on vaccine and vaccination programs [1]; concerned cost of chickenpox and of complications but not specifically AC-HAP [4]; reported only clinical data [1]. In literature the HAP for varicella may vary, depending on the study, ranging from 600 dollars to 4583 dollars per person, depending on the study [12–18].

The annual direct cost of varicella-related hospitalization of previously health children was estimated at 11210 dollars in Turkey and of 116,287 euro in Spain [12, 19]. Considering both inpatients and outpatients, an estimated annual cost of varicella may be even higher, accounting from € 187,5 million per year to 23,954,617 CAD [1, 20].

Neurological complications had been associated with high HAP of 6612 dollars. As well as in our study, the day cost of hospitalization contributed to more than 97% to the total cost of HAP [20]. We found that the median cost of varicella AC HAP was of 5366 euro, with an average annual cost of 23,252 €. To the best of our knowledge, no scientific studies on the economic burden of AC-HAP had been previously performed. A limitation of our study is the absence of evaluation of societal costs, the so called indirect costs, attributable to AC-HAP in children affected by varicella. The indirect costs, such as work and productivity loss incurred by parents caring for their children, and instructed medical and paramedical assistance, certainly account for an important part of the global costs.

Post marketing surveillance in the USA have consistently shown nearly 100% protection against moderate to severe varicella disease [21]. A scenario of no varicella vaccination compared to different scenarios with vaccination at 13 months of age evidenced an overall cost saving by a highly effective vaccine coverage [22]. In our study, none of the patients had been previously vaccinated for varicella, even if they were all eligible for vaccination (the youngest patient was 1.5 years old). Vaccination would have probably prevented varicella and its AC complication, avoiding vaccination in Italian children born from 1st January 2017. It is expected a reduction of the incidence of varicella and cases requiring hospitalizations: this could be cost-effective reducing hospital and medical care resources within a few years following higher vaccination rates. In particular, considering other Countries experience, mandatory vaccination programs are related to higher coverage rates and reductions in varicella-associated costs [23, 24]. Further studies could be performed to analyze these aspects in our population within a few years. Simulation analysis on societal and health costs revealed net costs saving with programs for routine varicella vaccination directed at children [25]. In details, a saving of 5.40 dollars for every dollar spent on routine vaccination of preschool children have been demonstrated [26]. Finally, a two-dose routine varicella vaccination of infants is estimated to reduce the costs-both direct and indirect- by 64% [27].

## Conclusions

Our study provides information regarding the financial burden of AC HAP in otherwise healthy children affected by varicella. AC complication may cause a long hospital stay and consequently a high direct sanitary cost. In the absence of published cost-of-illness reports investigating the burden of AC varicella in children, our results contribute to extend the knowledge on the economic impact of varicella disease. Hospital costs are an important end-point in health economic evaluation of the diseases and may be used by policy-maker decisors to implement vaccination programs.

Of note, no patient included in the study had received at least one dose of varicella vaccine. Prevention of varicella through vaccination is a priority to avoid the significant burden of its incidence and complications and should be encouraged from a both medical and economic viewpoint.

## Data Availability

Availability of data and material at Bambino Gesù Children Hospital, Dr. Bozzola’s repository.

## Abbreviations

VZV: Varicella zoster virus
AC: Acute cerebellitis
HAP: Hospitalization in the acute phase
